# Characterization of the basement membrane in kidney renal clear cell carcinoma to guide clinical therapy

**DOI:** 10.3389/fonc.2022.1024956

**Published:** 2022-11-10

**Authors:** Xi Xiong, Chen Chen, Jun Yang, Li Ma, Xiong Wang, Wei Zhang, Yuan Yuan, Min Peng, Lili Li, Pengcheng Luo

**Affiliations:** ^1^ Department of Urology, Wuhan Third Hospital, School of Medicine, Wuhan University of Science and Technology, Wuhan, China; ^2^ Department of Urology, Wuhan Third Hospital, Wuhan, China; ^3^ Department of Pharmacy, Wuhan Third Hospital, Wuhan, China; ^4^ Department of Urology, Wuhan Third Hospital and Tongren Hospital of Wuhan University, Wuhan, China; ^5^ Department of Oncology, Renmin Hospital of Wuhan University, Wuhan, China; ^6^ Central Laboratory, Renmin Hospital of Wuhan University, Wuhan, China

**Keywords:** kidney renal clear cell carcinoma, basement membrane, prognosis, tumor microenvironment, immunotherapy

## Abstract

**Background:**

Renal cell carcinoma (RCC) is the most common kidney cancer in adults. According to the histological features, it could be divided into several subtypes, of which the most common one is kidney renal clear cell carcinoma (KIRC), which contributed to more than 90% of cases for RCC and usually ends with a dismal outcome. Previous studies suggested that basement membrane genes (BMGs) play a pivotal role in tumor development. However, the significance and prognostic value of BMGs in KIRC still wrap in the mist.

**Methods:**

KIRC data were downloaded from the Gene Expression Omnibus (GEO) and The Cancer Genome Atlas (TCGA) databases. A prognostic risk score (PRS) model based on BMGs was established using univariate and least absolute shrinkage and selection operator (LASSO) and the Cox regression analysis was performed for prognostic prediction. The Kaplan-Meier analysis, univariate Cox regression, multivariate Cox regression, receiver operating characteristic (ROC) curves, nomogram, and calibration curves were utilized to evaluate and validate the PRS model. All KIRC cases were divided into the high-risk score (HRS) group and the low-risk score (LRS) group according to the median risk scores. In addition, single-sample gene set enrichment analysis (ssGSEA), immune analysis, tumor microenvironment (TME) analysis, principal component analysis (PCA), and half-maximal inhibitory concentration (IC50) were also applied. Expression levels of BMGs were confirmed by qRT-PCR in both human renal cancer cell lines and tissues.

**Results:**

We established the BMGs-based prognostic model according to the following steps. Within the TCGA cohort, patients’ prognosis of the HRS group was significantly worse than that of the LRS group, which was consistent with the analysis results of the GEO cohort. PCA patterns were significantly distinct for LRS and HRS groups and pathological features of the HRS group were more malignant compared with the LRS group. Correlation analysis of the PRS model and TME features, such as immune cell scores, stromal cell scores, and ESTIMATE values, revealed a higher immune infiltration in the HRS group compared with the LRS group. The chemotherapeutic response was also evaluated in KIRC treatment. It showed that the HRS group exhibited stronger chemoresistance to chemotherapeutics like FR-180204, GSK1904529A, KIN001-102, and YM201636. The therapeutic reactivity of the other 27 chemotherapeutic agents was summarized as well. Furthermore, the FREM2 level was measured in both human kidney tissues and associated cell lines, which suggested that lower FREM2 expression prompts a severer pathology and clinical ending.

**Conclusions:**

Our study showed that KIRC is associated with a unique BMG expression pattern. The risk scores related to the expression levels of 10 BMGs were assessed by survival status, TME, pathological features, and chemotherapeutic resistance. All results suggested that FREM2 could be a potential candidate for KIRC prognosis prediction. In this study, we established a valid model and presented new therapeutic targets for the KIRC prognosis prediction as well as the clinical treatment recommendation, and finally, facilitated precision tumor therapy for every single individual.

## Background

Renal cell carcinoma (RCC) is a type of urinary cancer contributing to approximately 2.4% of all types of cancers (1). It has high mortality and recurs easily. Clear cell renal cell carcinoma (ccRCC), papillary renal cell carcinoma (pRCC), and chromophobe renal cell carcinoma (chRCC) occupied ratios of 90%, 6% to 15%, and 2% to 5% of all renal cancers, respectively. The mainstream therapies for KIRC contains surgical resection, chemotherapy, and radiotherapy, however, all treatments showed limited effects (2).

Basement membranes (BMs) is a unique form of extracellular matrix (ECM) and acted just like a barrier for restraining cancer cells’ propagation to a distant place (3). Dynamic remodeling of the ECM usually participates in cancer development (4). The altered tumor microenvironment (TME) promotes tumor growth by pathologically remodeling ECM (5). BMs contain multiple components that determine the histological morphology framework, function in stress adaptation, and selective permeation (6). A large number of BM genes (BMGs) and their related mutations have been proved to be involved in multiple human diseases (7, 8). The TME, including various cellular components, ECM, and soluble growth factors, is highly related to tumor progression (9). The BMs is also an important histological boundary to distinguish the non-invasive (carcinoma *in situ*) and invasive tumors (10). BMs damage exacerbates local metastasis and the invasion of tumor cells (11).

Due to the noticeable function of BMs in tumor development, exploration of BMs-associated biomarkers and potential therapeutic targets to inhibit tumor progression has remarkably attracted researchers’ attention. For instance, the epithelial-mesenchymal transition of the BMs enables cells with an epithelial phenotype to transform to a quasi-mesenchymal phenotype, which could promote tumor metastasis from the initial location to distant organs (12). Collagen, laminin, and integrins are major components of bone marrow that contribute to tumor cell metastasis, and they are considered important anticancer targets (12). Also, cell migration could be strictly regulated by BMs, of which the breakdown is also a crucial step for allowing tumor progression (13).

In this article, a comprehensive evaluation of 541 KIRC datasets and 72 normal kidney tissue datasets was performed, and a BM-dependent prognostic risk score (PRS) model was developed. The PRS model is capable of independently predicting KIRC patients’ outcomes, and their chemotherapeutic resistance to FR-180204, GSK1904529A, KIN001-102, and YM201636 as well. In addition, we assessed its predictive value, diagnostic efficacy, chemotherapeutic effect, immunotherapeutic effect, and tumor immune infiltration in KIRC patients. These findings provide new insight into the involvement of the BMs in the therapy of KIRC.

## Materials and methods

### Microarray datasets

**Figure 1:**
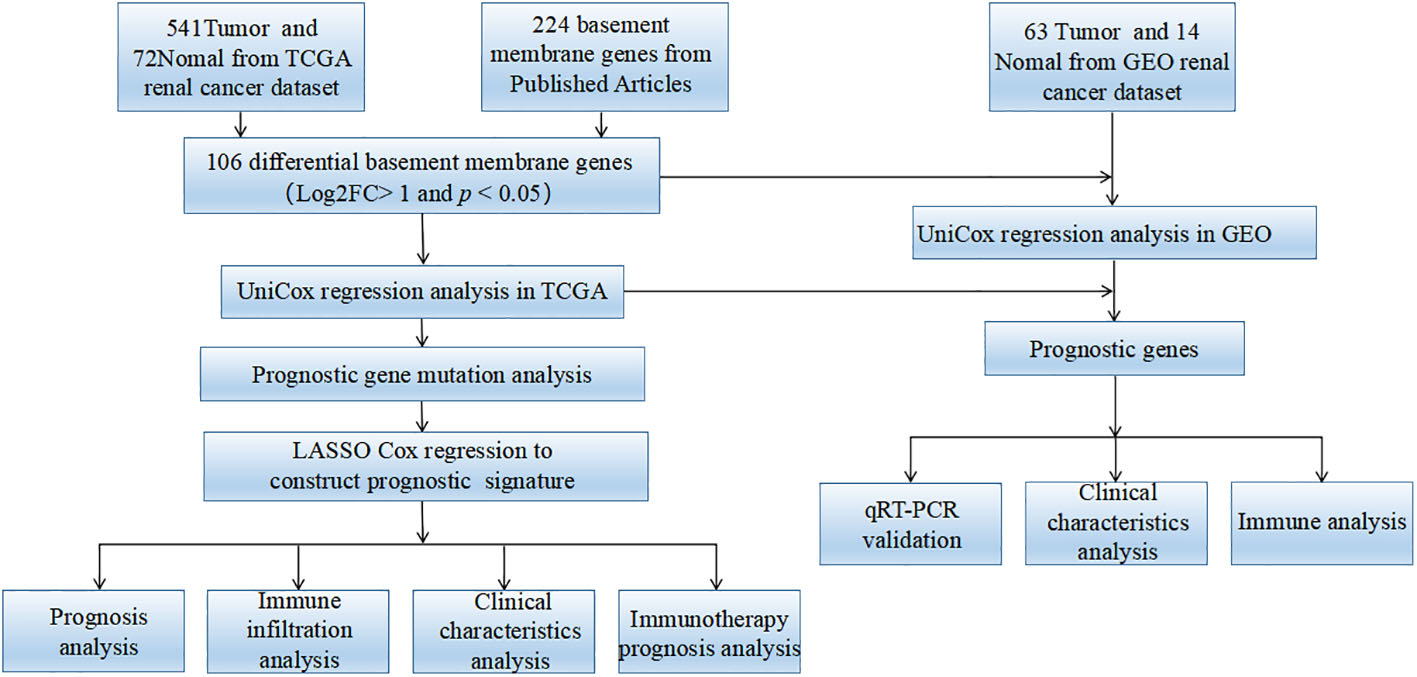
The flow diagram of this research.

The study flowchart is shown in **Figure 1**. We extracted RNA-seq datasets from the TCGA database (14), involving 541 KIRC samples and 72 normal kidney samples, as well as clinical data of 537 KIRC cases retrieved from the TCGA database. The GEO database was utilized to access microarray data distribution for GEO: GSE167573 *via* the GPL20795 platform (15). Using an annotation platform, it was attempted to transform the Entrez gene ID of each sample to the associated gene symbol. In the case of targeting the same Entrez gene ID *via* multiple probes, the average value was used. A combination of individual RNA-seq data was carried out by sample ID using Perl.

### Acquisition of BMGs

In a recent study, 224 BMGs were identified (7). The expression levels of 224 BMGs were then extracted from TCGA and GEO cohorts [FDR< 0.05, log2 (fold-change (FC)] > 1]. The “limma” R package was used to identify differential expressed genes (DEGs). Additionally, 106 BMGs with common differences between TCGA and GEO cohorts were selected.

### Identification of genes with differential expression and functional enrichment

The “limma” R package was utilized for the analysis of the differential expression of BMGs in cancerous and normal tissue specimens. It was attempted to consider the statistical significance of genes with FDR< 0.05. The “org.Hs.eg.db” R package was utilized, in order to transform each DEG’s gene symbol into an Entrez Gene ID. Using the “clus-terProfiler” R package, KEGG pathway enrichment and GO analyses were carried out on DEGs to figure out the primary biological characteristics and cell functioning pathways (P< 0.05). Finally, we employed the “enrichplot” and “ggplot2” R packages to illustrate the results of the enrichment analyses.

### Construction and validation of a PRS model

Samples attained from TCGA and those samples from GSE167573 were designated as training datasets and test datasets, respectively. Using the ID of each sample, it was attempted to combine the expression levels of differentially expressed BMGs of each sample with the relevant prognostic outcomes. Prognosis-associated genes (PAGs) were identified using univariate Cox regression (uni-Cox-reg) on differentially expressed BMGs from the TCGA (training dataset) and GEO (test dataset). The “maftools” R package was applied for the analysis of the mutation and the associated genes in the training dataset of KIRC samples. The analysis of PAGs was performed *via* the “glmnet” R package with the assistance of the least absolute shrinkage and selection operator (LASSO), which develop a PRS model that could accurately predict overall survival rate (OS) of KIRC samples. The estimation of the penalty parameter of the model was carried out *via* the 10-fold cross-validation. The following formula was utilized for calculating the risk score of each sample: risk score $\sum_{i=1}^{n}coef\;(i)\;\times \;expr\;(i)\;$ , in which “expre” represents the gene expression levels from the PRS model, and “coef” indicates non-zero regression coefficients derived by LASSO regression analysis (**Supplementary Table 2**). Division of samples into high-risk score (HRS) group and low-risk score (LRS) group was carried out using the median value of risk scores. The log-rank test and Kaplan-Meier analysis were utilized to compare OS-related differences between the two above-mentioned groups. The “survivalROC” R package was utilized to plot the time-dependent ROC curves, which assisted in investigating the accuracy of predictability of the PRS model. The validity and accuracy of the PRS model were verified *via* the test dataset.

### Principal component analysis

With the assistance of the “limma” R package, PCA of the PRS model and differentially expressed BMGs from the TCGA were conducted to compare the two above-mentioned groups. First, using PCA, expression patterns of all the differentially expressed BMGs were assessed. Then, the expression patterns of BMGs attained from the PRS model were assessed using PCA. The PCA results were illustrated *via* the ggplot2 R package.

### Association of risk scores with clinical characteristics

In the TCGA cohort, using the sample ID, we incorporated clinical features with the risk score of each sample. The association between risk scores and clinical features was investigated by the “limma” R package. It was attempted to collect KIRC-related clinical data from the GEO cohort to analyze the association between risk scores and clinical features. Clinical features between two or more groups were compared *via* Kruskal-Wallis and Wilcoxon rank-sum tests. P< 0.05 indicated a significant difference.

### Gene set variation analysis

The “GSVA” R package was employed to compare differences in biological processes between the two above-mentioned groups. Assessment of pathway changes or biological processes is feasible *via* GSVA, owing to its non-parametric and unsupervised features, *via* expression matrix samples (16). The “c2.cp.kegg.v7.4.symbols” was obtained from the GSEA database (https://www.gsea-msigdb.org/gsea/msigdb) (17). A statistically significant enrichment pathway was demonstrated by FDR<0.05.

### Predicting potential compounds for the treatment of KIRC

The pRRophetic R package was employed to predict the IC50 of common chemotherapeutic drugs (18). IC50 indicates a substance’s efficacy in terms of inhibiting particular biochemical or biological functions. We employed Wilcoxon signed-rank test to assess group differences. Using the “pRRophetic”, “limma”, “ggpub”, and “ggplot”2 R packages, compounds that could be used for KIRC treatment were predicted.

### GO and KEGG pathway enrichment assays

Differentially expressed BMGs in cancerous and normal tissue specimens were screened. Through the “clusterProfiler” R package, GO and KEGG enrichment analyses of these genes were performed (19).

### Estimation of TME

The ssGSEA was conducted using the “GSEABase” and “GSVA” R packages to assess immune-associated infiltration in each TCGA cohort sample. Moreover, the gene sets were taken from the previous work to evaluate immune-associated characteristics in TME, including numerous human immune-associated functions and immune cell subtypes, such as regulatory T cells (Tregs), NK T cells, CD8^+^ T cells, etc (**Supplementary Table 3**) (16, 20). The difference in the enrichment scores between the LRS and HRS groups was analyzed *via* the ssGSEA algorithm. The association between immune cells and risk scores was predicted using “TIMER”, “EPIC”, “MCPcounter”, “QUANTISEQ”, “CIBERSORT”, “XCELL”, and “CIBERSORT” to assess immune infiltration status (21). P< 0.05 indicated statistical significance.

### Cox regression analysis and nomogram development

We constructed nomograms containing clinical features and PRS models through the “rms” R package to predict the OS of KIRC specimens on the basis of the TCGA cohort. Prediction of the accuracy of the nomogram was conducted *via* time-dependent calibration curves. Using multivariate Cox regression (multi-Cox-reg) analysis, we determined whether the PRS model was an independent indicator of OS in KIRC. ROC curves were utilized to calculate the AUC value, which showed the diagnostic value of the nomogram.

### Patients’ recruitment and ethical statement

We recruited 6 patients with kidney cancer who received partial and radical nephrectomy at Wuhan Third Hospital from August 2021 to August 2022. The protocol passed the approval of the Ethics Committee of Wuhan Third Hospital. All patients were diagnosed with renal cell carcinoma.

### Cell culture

HK-2, ACHN, and CAKI cell lines were attained from a company (BNCC, Henan, China). The culture of HK-2, ACHN, and CAKI cell lines in DMEM-F12 and RPMI-1640 was undertaken at 37°C media with 1% streptomycin-penicillin and 10% FBS (Gibco).

### RNA extraction and qRT-PCR

RNA isolation kit (R6934-01, Omega Bio-Tek, USA) was attempted to extract total RNA from the three above-mentioned cell lines on the basis of the manufacturer’s protocol. TOYOBO ReverTra Ace qPCR RT kit was utilized for reverse transcription. A Bio-Rad CFX Manager system was employed to carry out real-time PCR. The FREM2 expression level was calculated using the 2−ΔΔCq method (22). The primers are presented in **Table 1**.

**Table 1:** Primers designed for qRT-PCR.

Gene symbol	Forward primer (5’-3’)	Reverse primer (5’-3’)
FREM2	ACTCAGTTCACACAAGCTGACA	TCCATGCCCAATTCAGACGA
β-actin	CCTGGCACCCAGCACAAT	GGGCCGGACTCGTCATAC

### Statistical analysis

Spearman correlation analysis was employed to calculate correlation coefficients. Using the Kaplan–Meier method, the Cox regression model, and the log-rank test, the prognostic value was evaluated. The Wilcoxon rank-sum test was utilized to compare the two groups. Three or more groups were compared by the Kruskal-Wallis test. Two-tailed P< 0.05 indicated a significant difference. R 4.2.0 software was utilized to process data statistically.

## Results

### Enrichment analysis of samples

**Figure 2:**
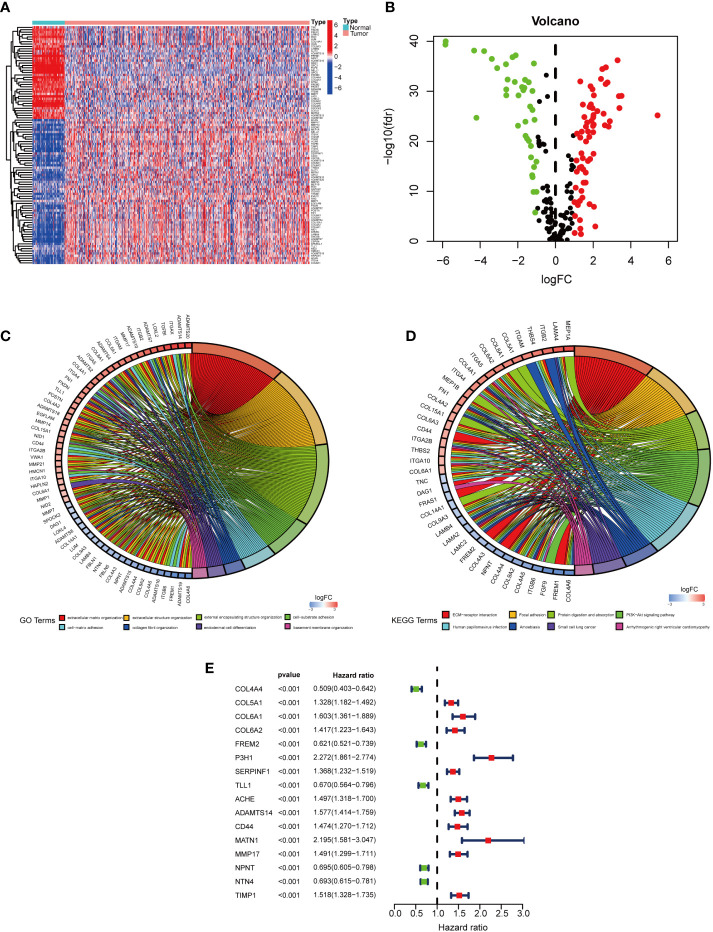
Differentially expressed BMGs in KIRC cases. **(A)** Enrichment plot of 106 BMGs in normal and tumor tissues. **(B)** The volcano plot of 106 differentially expressed BMGs with differential expression. **(C, D)**. Noticeable enrichment of GO terms and KEGG pathways of 106 BMGs. **(E)** The forest plot illustrated a uni-Cox-reg analysis of the association of 16 BMGs with prognosis.

The expression levels of BMGs in cancerous and normal samples were compared. 106 differentially expressed BMGs in KIRC tissue samples were obtained, involving 67 upregulated and 39 downregulated differentially expressed BMGs (**Figures 2A, B**). The differentially expressed BMGs are listed in **Supplementary Table 1**. The results of Gene Ontology (GO) enrichment analysis revealed that ECM tissues, collagen-containing ECM, and ECM structural components were highly enriched for GO terms (**Figure 2C**). The results of the Kyoto Encyclopedia of Genes and Genomes (KEGG) pathway enrichment assay indicated that ECM receptor interaction terms were highly enriched (**Figure 2D**). Collectively, the role of BMGs in the KIRC progression is noteworthy.

### Development and validation of the PRS model

**Figure 3:**
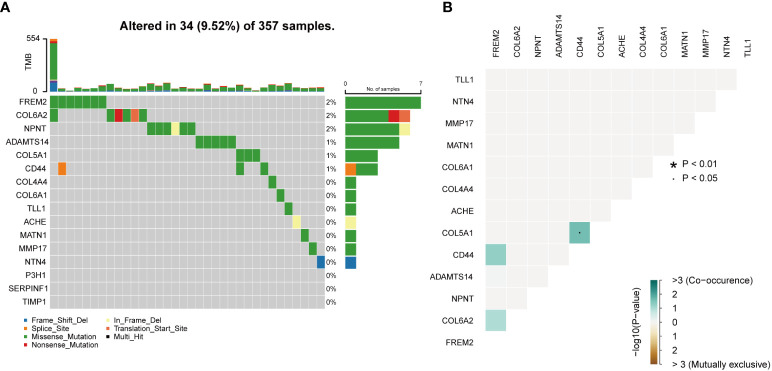
Mutations in BMGs. **(A)** The mutation frequency of 16 BMGs in 357 KIRC cases from the TCGA cohort. **(B)** The analyses of mutational co-occurrence and exclusion for 16 BMGs.

**Figure 4:**
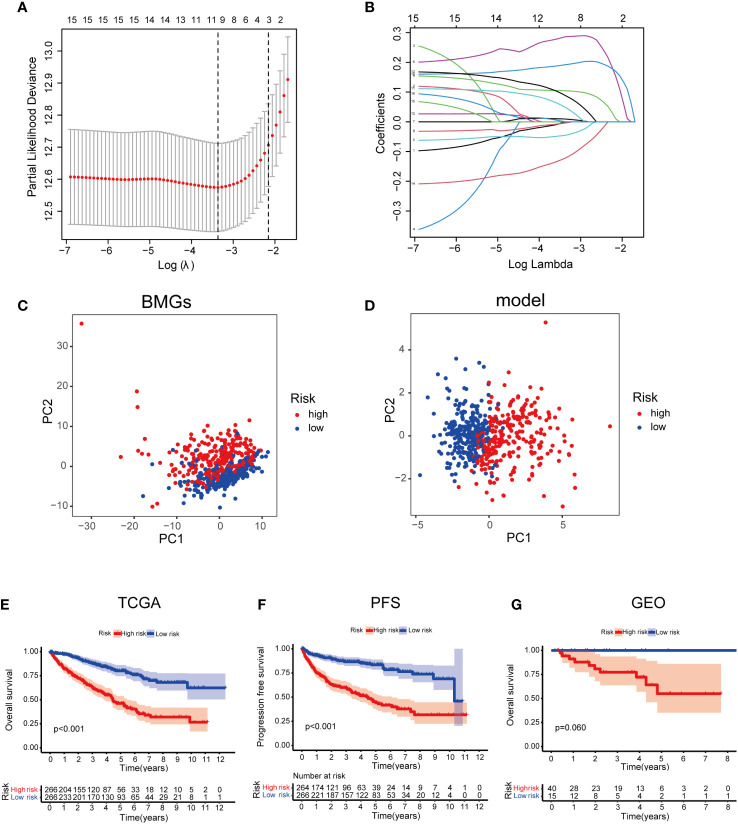
Developing a PRS model using 10 BMGs. **(A)** Identification of 10 BMGs for a PRS model. **(B)** The LASSO coefficients of the 10 BMGs. **(C)** PCA on the basis of BMGs in KIRC. **(D)** PCA on the basis of risk scores to discriminate low- and high-risk score patients in the TCGA cohort. **(E)** Kaplan–Meier survival curves of OS in LRS and HRS groups in the GEO cohort. **(F)** Kaplan–Meier survival curves of OS in LRS and HRS groups in the TCGA cohort. **(G)** The comparison of PFS between LRS and HRS groups in the TCGA cohort.

In the TCGA cohort, uni-Cox-reg analysis was employed for the analysis of 106 differentially expressed BMGs, of which 16 PAGs were identified (P< 0.05) (**Figure 2E**). The profile of somatic mutation of the 16 prognosis-associated BMGs was analyzed. It was revealed that 34 of 357 KIRC samples had mutations in BMGs, as indicated in **Figure 3A**, with a frequency of 9.52%. FREM2 had a higher frequency of mutations than COL6A2. P3H1, SERPINF1, and TIMP1 showed no mutations in KIRC samples. Further analysis revealed a mutation association between CD44 and FREM2, COL6A2 and FREM2, and COL5A1 and CD44 (**Figure 3B**). LASSO regression analysis was employed to reduce the number of genes studied. Ten genes (COL4A4, FREM2, P3H1, SERPINF1, TLL1, ACHE, ADAMTS14, CD44, MMP17, and NPNT) were used in the development of a PRS model (**Figures 4A, B**). The following formula was utilized for calculating the risk score: COL4A4 × (-0.000332379306699992) + FREM2 × (-0.0453284014970285) + P3H1 × (0.286241547319786) + SERPINF1 × (0.00925436343813996) + TLL1 × (-0.0039265754833404) + ACHE × (0.104431990160361) + ADAMTS14 × (0.192192783740491) + CD44 × (0.0481638351937017) + MMP17 × (0.0880107569563803) + NPNT × (-0.121725611636459) (23), which was exhibited in **Supplemental Table 2**. The PRS model developed to distinguish between high- and low-risk KIRC samples is illustrated in **Figures 4C, D**.

### Risk scores and clinical characteristics

**Figure 5:**
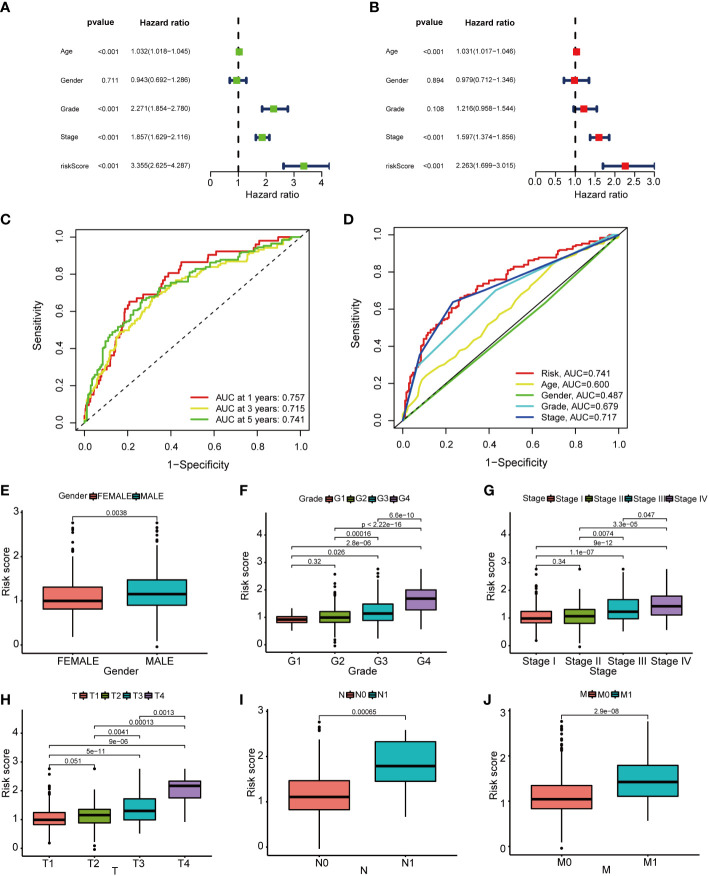
The prognostic value of the basement membrane score combined with pathological features in the TCGA cohort. **(A, B)**. The multi-Cox-reg and uni-Cox-reg analyses of the risk score and clinical variables in association with overall survival. **(C, D)**. The 1-, 3-, and 5-year ROC curves were plotted to indicate the association of the risk scores with clinical characteristics. **(E–G)**. The relationship of pathological features [that’s gender **(E)**, grade **(F)**, TNM stage **(G)**, tumor invasion **(H)**, lymphoid metastasis **(I)**, and distal metastasis **(J)**] with risk scores.

On the basis of the median value of the risk scores of the samples in the training dataset, the division of the risk scores of the samples into the LRS group (n = 266) and HRS group (n = 266) was carried out. In the TCGA cohort, a poorer prognosis was identified in the HRS group compared to the LRS group (P = 0.001; **Figure 4E**). Additionally, in TCGA cohort, the progression-free survival (PFS) was worse in the HRS group than that in the LRS group (**Figure 4F**). To validate the accuracy of the PRS model, the samples from the GSE167573 were divided into the LRS group (n = 15) and the HRS group (n = 40). Based on the median values achieved from the TCGA. In the GSE167573, since a worse prognosis was found in the HRS group than that in the LRS group, it was shown that the PRS model predicted OS in cases with KIRC (**Figure 4G**). On the basis of the results of univariate and multivariate analyses, age, pathological stage, and risk score could independently predict OS (**Figures 5A, B**). The PRS model was verified by plotting 1-, 3-, and 5-year receiver operating characteristic (ROC) curves (**Figure 5C**). The results of the area under the ROC curve indicated that the risk score (AUC=0.741) had a better prognostic value than the other indicators (**Figure 5D**). We further analyzed the correlation of risk scores with gender, age, grade, American Joint Committee on Cancer (AJCC) TNM stage and pathological stage in clinical samples (24). However, there was no statistically significant relationship between risk score and age (**Supplementary Figure 1A**). Compared to women, men had higher risk scores (**Figure 5E**). Risk scores related to grade and advanced pathological stages, including AJCC-T (tumor invasion), AJCC-N (lymphoid metastasis), and AJCC-M (lymphoid metastasis) stages (distal metastasis) (**Figures 5F–J**).

### Construction of nomogram

**Figure 6:**
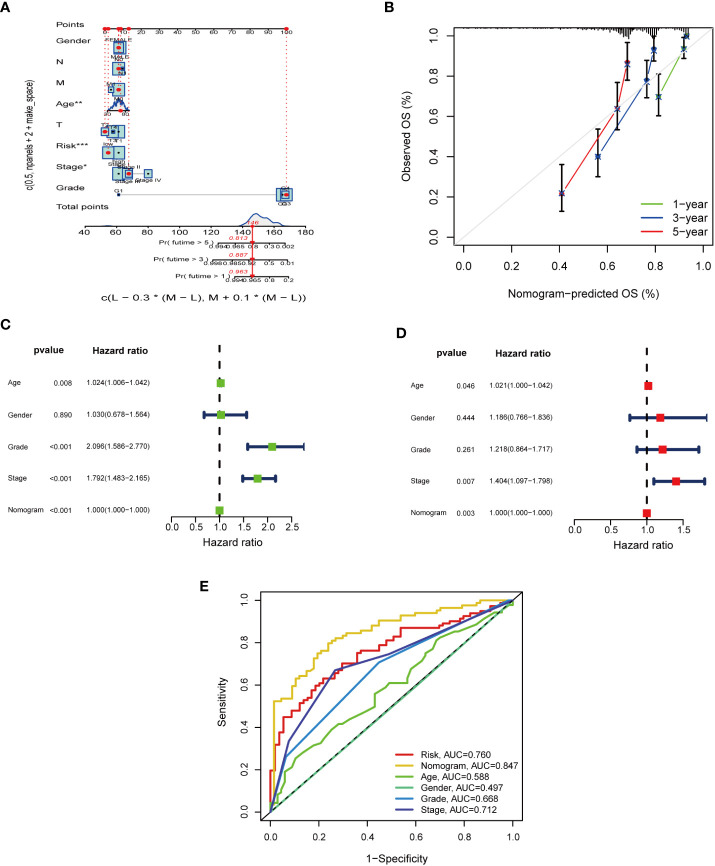
The predictive value of nomogram design and nomogram scores when combined with pathological features in the TCGA cohort. **(A)** Nomogram predicting 1-, 3-, and 5-year OS in the TCGA cohort. **(B)** The calibration curves for 1-, 3-, and 5-year OS. **(C, D)**. The multi-Cox-reg and uni-Cox-reg analyses of the nomogram and pathological features. **(E)** ROC curves for BM scores, nomogram, and pathological features.

To evaluate the sensitivity and specificity of the model for prognosis. We performed a nomogram *via* incorporation of age, gender, pathological stage, grade, and PRS for the purpose of predicting OS in KIRC samples (**Figures 6A, B**). On the basis of the findings of uni-Cox-reg and multi-Cox-reg analyses, the nomogram model, age, and pathological stage were independent prognostic factors (**Figures 6C, D**). Based on the 1-, 3-, and 5-year calibration curves, the nomogram could accurately predict OS in cases with KIRC (**Figure 6E**). The AUC values indicated that the predictability of nomogram (AUC= 0.847) was greater compared to age (AUC=0.588), pathological stage (AUC=0.712), grade (AUC=0.688), and PRS model (AUC=0.760).

### Immune-associated characteristics

**Figure 7:**
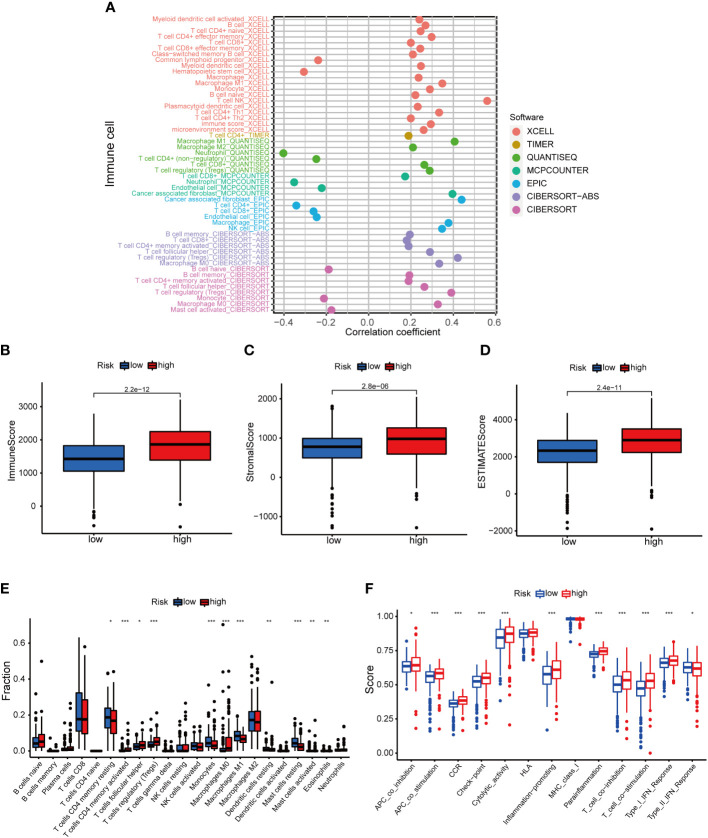
The role of basement membrane model in the immunotherapy. **(A)** The immune cell bubble of risk groups. **(B–D)**. Immune-related scores in LRS and HRS groups. **(E, F)**. The relative proportions of immune cells and immunological activities were assessed using ssGSEA in the HRS and LRS groups. *p< 0.05, **p< 0.01, and ***p< 0.001.

**Figure 8:**
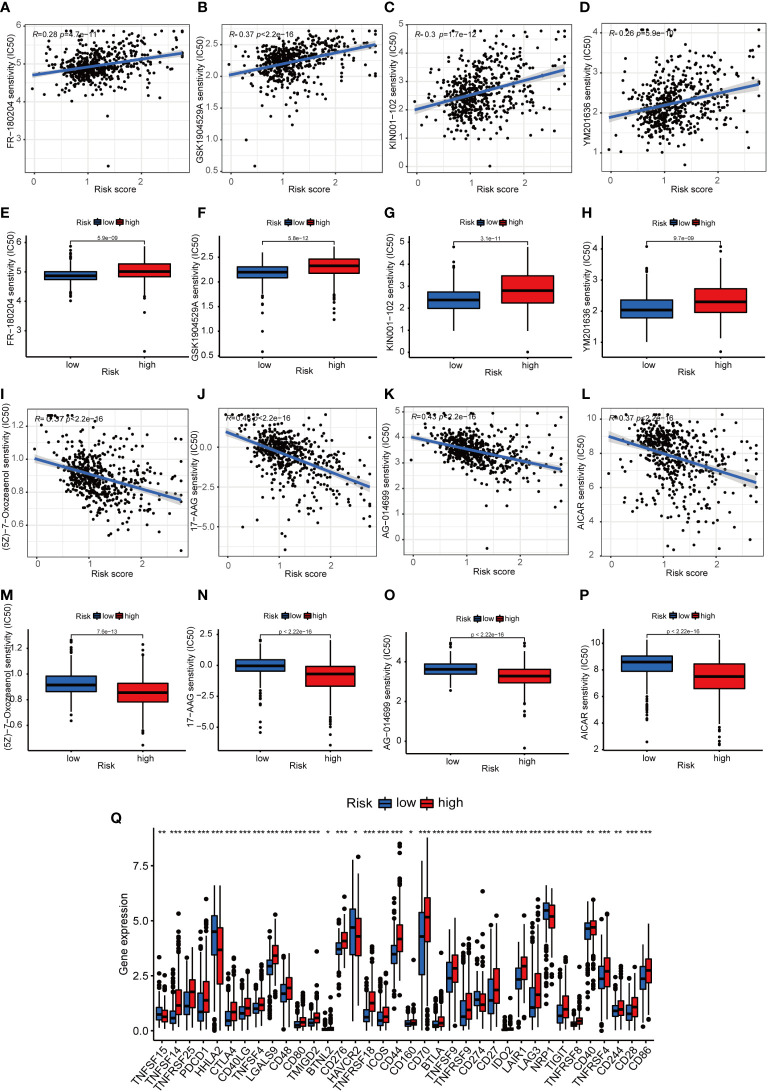
The immunotherapeutic prognosis for risk-dependent groups. **(A–D)**. The negative association of patients’ risk scores with IC50 values for chemotherapeutics. **(E–H)**. IC50 values for chemotherapeutics in the HRS group were higher compared to the LRS group. **(I–L)**. The negative association of patients’ risk scores with the IC50 values of chemotherapeutics. **(M–P)**. The IC50 values of chemotherapeutic agents were lower in the HRS group compared to the LRS group. **(Q)**. The expression levels of 18 checkpoints in various risk categories. *p< 0.05, **p< 0.01, and ***p< 0.001.

The positive association of risk score with NK T cells, cancer-associated fibroblasts, Tregs, M1 macrophages, CD4 + T cells, Th1 cells etc. was shown in the resutls. The risk score was negatively associated with neutrophils, CD4+ T cells, and CD8+ T cells (P< 0.05) (**Figure 7A**, **Supplementary Table 3**). The HRS group showed higher immune cell scores, stromal cell scores, and ESTIMATE values, indicating a higher immunological infiltration level in the HRS group (**Figures 7B–D**). It was suggested that there was a different TME in the HRS and LRS groups. The ssGSEA revealed a relatively higher proportion of immune cells and immune function in the HRS group (**Figures 7E, F**). The majority of the immune checkpoints were significantly activated in the HRS group (**Figure 8Q**), thus, individualized immunosuppression was suggested for the treatment of cases with KIRC (25).

### Prediction of patients’ response to chemotherapy

The association of risk scores with poor prognosis was confirmed, and we further investigated the correlation between chemoresistance and risk scores. Half of the maximum inhibitory concentration (IC50) of the chemotherapy in the TCGA cohort was predicted using the “pRRophetic” R package (26). Samples with a high-risk score were insensitive to chemotherapeutic drugs, such as FR180204, GSK1904529A, KIN001102, and YM201636 (**Figures 8A–H**). Except for four chemotherapy drugs (FR-180204, GSK1904529A, KIN001-102, and YM201636) that had higher IC50 values in the HRS group (**Figures 8I–P**), the remaining 27 chemotherapy drugs had lower IC50 values in the HRS group (**Supplementary Figure 2**) and risk score negatively correlates with chemotherapy resistance (**Supplementary Figure 3**). Therefore, patients’ responses to most of the chemotherapy drugs in the HRS group were superior to those in the LRS group.

### GSVA

To investigate the differences in biological functions between low- and high-risk groups, we performed GSVA enrichment analysis. The “c2.cp.kegg.v7.4” gene sets were available in the Molecular Signatures Data Base (MSigDB), and they were utilized in GSVA for the analysis of the biological behaviors in the two above-mentioned groups. Most metabolic pathways, e.g, fatty acid metabolism and propanoate metabolism, would be remarkably enriched in the LRS group (**Supplementary Figure 1B**). A negative correlation of risk scores with metabolic pathways was confirmed. There was no significant enrichment of pathways in the HRS group.

### Verification of prognostic BMGs in the GEO and TCGA cohorts

**Figure 9:**
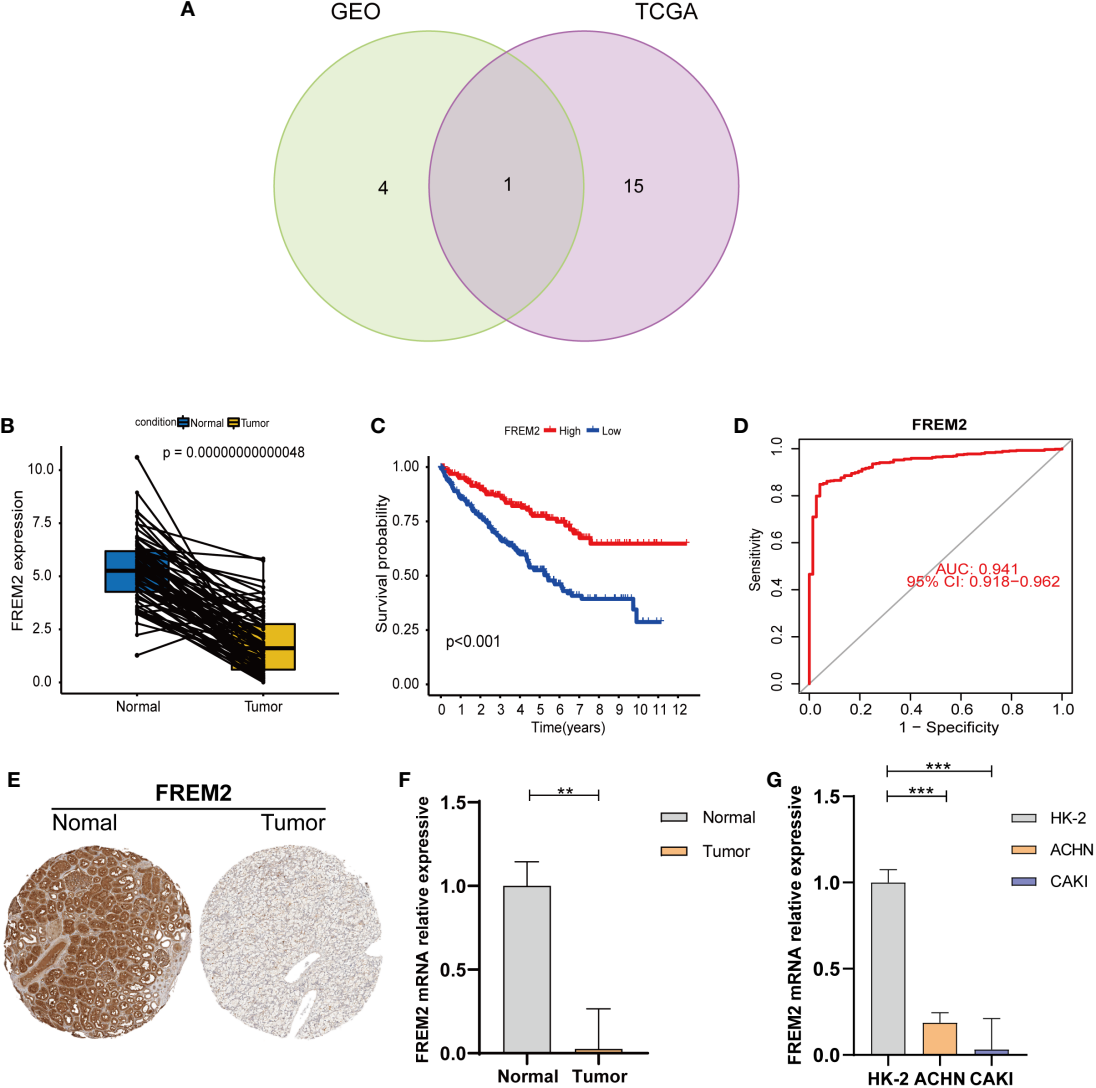
The prognostic BMGs were verified by uni-Cox-reg analysis. **(A)** Uni-Cox-reg analysis and the intersection of BMGs in the TCGA and GEO cohorts. **(B)** Paired differentiation analysis of FREM2 expression in cancerous and normal specimens from the same patient (*P* = 0.00000000000048, by the WRS test). **(C)** Various FREM2 expression levels were utilized to carry out survival analysis. Patients were clarified into high or low expression levels on the basis of the median expression level (*P*< 0.001 by the log-rank test). **(D)** ROC curves were plotted to evaluate the FREM2’s most accurate predicting ability. **(E)** Immunohistochemistry of FREM2 in normal and tumor tissues of the kidney. **(F)** The qRT-PCR findings of FREM2 in cancerous and normal kidney specimens (*P<* 0.01). **(G)** The qRT-PCR findings of FREM2 in renal cell lines. **p< 0.01 and ***p< 0.001.

**Figure 10:**
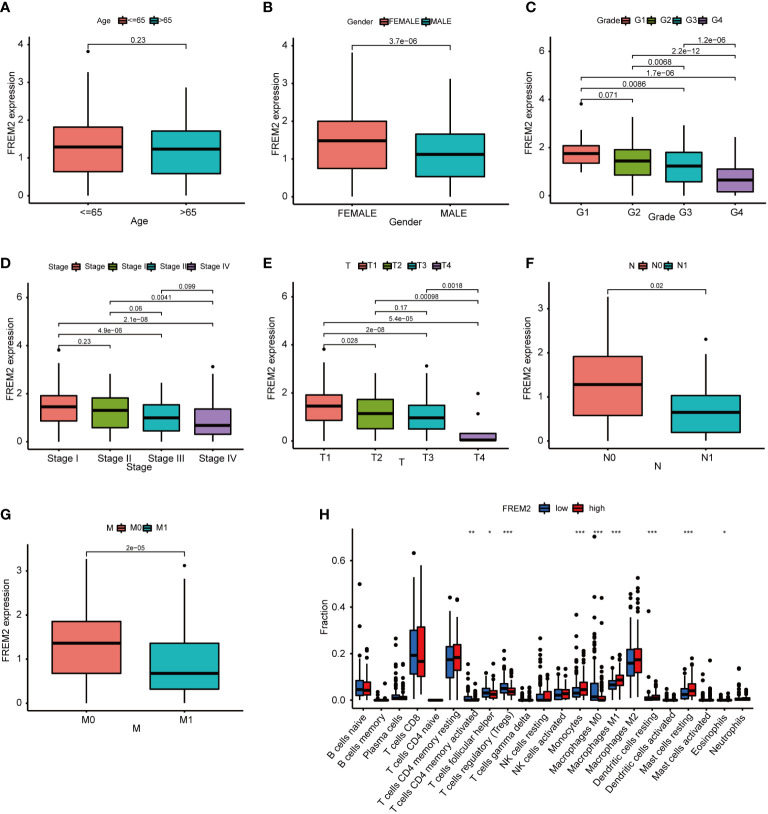
The relationship between FREM2 expression level and pathological features, as well as immune cell in patients with high and low FREM2 mRNA expression. **(A–G)**. The relationship of FREM2 expression level and clinicopathological features. **(H)** The relative proportion of immune cells using ssGSEA in patients with high and low FREM2 mRNA expression. *p< 0.05, **p< 0.01, and ***p< 0.001.

To investigate target genes in basement membrane genes that can be used as predictors of clinicopathology and prognosis. We analyzed prognostic BMGs in the TCGA and GEO cohorts by univariate cox regression (**Figure 2E**; **Supplementary Figure 4**), and plotted the Venn diagram to obtain the intersected BMGs (**Figure 9A**). The FREM2 expression level was reduced in cancerous tissues compared to normal tissues (**Figure 9B**). FREM2 expression levels remarkably differed between the HRS and LRS groups (P< 0.001). A noticeably higher survival rate was found in the HRS group, which was compatible with the expression level in the normal and cancerous tissues (**Figure 9C**). To further figure out the accuracy of the FREM2 gene in predicting renal cancer, the ROC curve of the FREM2 gene was plotted and AUC was 0.941, indicating that FREM2 could be utilized as a potential prognostic indicator for KIRC (**Figure 9D**). We obtained immunohistochemical data on FREM2 expression level in kidney cancer from the Human Protein Atlas database (27), We further verified the expression level of FREM2 in human kidney cancer and normal tissues by qRT-PCR (**Figures 9E, F**). In addition, we verified the FREM2 expression level in kidney cells by qRT-PCR, and the FREM2 expression level was higher in HK-2 normal kidney cells and lower in kidney tumor cells (ACHN and CAKI cell lines), which was basically consistent with the human tissue verification results (**Figure 9G**). In addition, FREM2 expression decreased as the tumor stage advanced (**Figures 10A–G**). FREM2 expression decreased and was associated with poor prognosis. TME immune cell infiltration differences between high and low FREM2 expression patients were explored using the FREM2 median expression value as the cutoff value. Tumors with low FREM2 expressions had significantly increased infiltration in Tregs than patients with high expressions (**Figure 10H**). Collectively, FREM2 may be a therapeutic target or biomarker for KIRC.

## Discussion

The BMs are necessary for the development of epithelial tissues and organs. Moreover, changes in the structural integrity and composition of the BMs are noticeably associated with the tumor development (28). In clinical practice, tumors located on the epithelial side of the BMs are considered as benign, while tumors penetrating the BMs are malignant, and thus, they may acquire the potential for metastasis (29). In order to metastasize, tumor cells invade *via* penetrating BMs, however, the precise mechanism indicating which tumor cells can penetrate BMs remains obscure. The two most abundantly expressed proteins in the BMs are collagen IV and laminin, of which collagen IV is the main structural backbone of BMs, while laminin facilitates cell signaling (30). It has been suggested that invasion of BMs is due to the action of proteases, and in particular, chemical degradation of BMs by matrix metalloproteinases (MMPs) occurs, while clinical trials on MMP inhibitors have failed to achieve outstanding results (31, 32). It has also been demonstrated that the stiffness of BMs is a critical determinant of tumor cells’ ability to penetrate BMs (3). Studies have noticeably concentrated on the function of a single BM component in tumors, whereas the exact role of BMGs in tumors is worthy of exploration. The function of BMGs in KIRC should be essentially unveiled to provide guidance for its clinical treatment. This is the first study to explore the association of BMGs with KIRC.

We first extracted 106 differentially expressed BMGs in the cancerous and normal kidney tissues, and these BMGs were utilized to estimate the PRSs in TCGA and GEO cohorts. We developed a PRS model for the prediction of OS in renal cancer in the TCGA cohort that would be advantageous to better figure out the significance of the BMGs. A difference was identified in survival between the HRS and LRS groups. The test dataset showed identical results, indicating the accurate prognostic function of the PRS model. On the basis of the results of the multivariate analysis, the PRS model and nomogram can be used as independent prognostic tools. In addition, ROC curves could be plotted the *via* incorporation of pathological features with the PRS model and the nomogram. The results revealed that the nomogram could more accurately predict patient survival.

To further find out the role of prognostic risk scoring models in KIRC, patients’ response to chemotherapeutic agents in the LRS and HRS groups was evaluated. A positive association of patients’ response to chemotherapeutic agents (FR−180204, GSK1904529A, KIN001−102, and YM201636) with risk scores was confirmed, and the remaining risk scores were negatively associated with 27 chemotherapeutic agents. Thus, cases in the HRS group were sensitive to most chemotherapeutic drugs and were chemo-resistant to a small number of chemotherapeutic drugs. A higher immune infiltration was also identified in the HRS group compared to the LRS group. The majority of immune checkpoint inhibitors (ICIs) were also more activated in the HRS group. Therefore, it is feasible to select appropriate ICIs for KIRC patients regrouped by risk patterns. In clinical practice, ICIs are an effective treatment for KIRC and other malignancies. However, only a few cases have a durable response to ICI therapy, indicating the necessity of providing individualized immunotherapy for such patients. In the present study, a higher proportion of suppressor T cells was identified in the HRS group, including Tregs and immune and inflammatory cells. CD8^+^ T cells are the immune cells that could be utilized for targeted cancer therapy. An immune checkpoint blocker, CTLA4, was significantly activated in the HRS group. CTLA4 is limited to the activated T cells and Tregs, and CTLA4 impedes tumor progression by depleting Tregs and modulating Treg suppressive activity (33), and therefore, anti-CTLA4 antibodies are indicated in patients with high risk scores. BMGs in KIRC significantly differed between the LRS and HRS groups.

To further explore the DEGs in the two above-mentioned groups, we identified the prognosis-related risk difference genes by the uni-Cox-reg model, and mapped the intersection of TCGA prognosis risk difference genes and GEO-related risk prognosis difference genes by the Venn diagram, and FREM2 was found to be essential. In this study, FREM2 has the highest mutation frequency among differentially expressed BMGs. FREM2 encodes a protein that is primarily localized to the BMs. This protein is critical for skin and renal epithelial integrity. It was previously shown that the high FREM2 expression in tumor tissues, and mutations in FREM2 exhibited an association with poorer prognosis of cancer patients (34). We further validated FREM2 expression level in human kidney tissues and cells, and found that the staging of pathological features was negatively correlated with the level of FEM2 expression. However, the present study also has some limitations. Our risk assessment data for basement membrane genes were obtained from public databases, and there is a lack of additional external transcriptomic information to validate the role of basement membrane genes in KIRC. the specific molecular mechanisms of basement membrane genes in KIRC are unknown, so further molecular experiments are needed. On the other hand, there were 72 normal samples and 541 tumor samples in our kidney samples. The heterogeneity of the samples may affect the accuracy of the data analysis.

## Conclusions

In summary, PRS for BMGs were positively correlated with clinical characteristics (age, gender, grade, and pathological stage). In addition, risk scores can predict patients’ sensitivity to chemotherapy. Therefore, risk scores and clinical stages directly guide the clinical management of patients. This study provided a valid model for predicting the prognosis of KIRC, as well as the clinical treatment, thus facilitating the development of individualized tumor therapy. According to human samples and *in vitro* research, FREM2 can be utilized as a prognostic biomarker for KIRC. However, it is advised to conduct additional research to figure out the role and mechanism of BMGs in KIRC patients and to develop new treatments.

## Data availability statement

The original contributions presented in the study are included in the article/**Supplementary Material**. Further inquiries can be directed to the corresponding authors.

## Ethics statement

The studies involving human participants were reviewed and approved by The Ethics Committee of Wuhan Third Hospital. The patients/participants provided their written informed consent to participate in this study. Written informed consent was obtained from the individual(s) for the publication of any potentially identifiable images or data included in this article.

## Author contributions

PL designed the project and supervised the study; XX and CC conducted the experiments and revised the article. LM and JY contributed to the data analysis and original draft preparation; XX, LL, and MP reviewed and edited the manuscript; WZ provided valuable suggestions for study; XW and YY edited the language. All authors contributed to the article and approved the submitted version.

## Funding

This study was supported by the national natural science foundation of China (81770688), Hubei leading talent program in medicine, and Wuhan application foundation and frontier project (2020020601012209).

## Conflict of interest

The authors declare that the research was conducted in the absence of any commercial or financial relationships that could be construed as a potential conflict of interest.

## Publisher’s note

All claims expressed in this article are solely those of the authors and do not necessarily represent those of their affiliated organizations, or those of the publisher, the editors and the reviewers. Any product that may be evaluated in this article, or claim that may be made by its manufacturer, is not guaranteed or endorsed by the publisher.

## Abbreviations

RCC, Renal cell carcinoma; KIRC, Kidney renal clear cell carcinoma; BMGs, Basement membrane genes; TCGA, The Cancer Genome Atlas; GEO, Gene Expression Omnibus; PRS, Prognostic risk score; LASSO, least absolute shrinkage and selection operator; ROC, receiver operating characteristic; HRS, high-risk score; ssGSEA, single-sample gene set enrichment analysis; TME, tumor microenvironment; PCA, principal component analysis; ccRCC, Clear cell renal cell carcinoma; chRCC, chromophobe renal carcinoma; BM, Basement membrane; ECM, extracellular matrix; WRS, Wilcoxon rank-sum; IACUC, Institutional Animal Care and Use Committee; multi-Cox-reg, multivariate Cox regression; PAGs, Prognosis-associated genes; uni-Cox-reg, univariate Cox regression; OS, Overall survival rate; GO, Gene Ontology; KEGG, Kyoto Encyclopedia of Genes and Genomes; MMPs, matrix metalloproteinases; ICIs, thimmune checkpoint inhibitors.
