# Supplementary material for: Characterization of the basement membrane in kidney renal clear cell carcinoma to guide clinical therapy
Source: Front Oncol. 2022 Nov 10;12:1024956. doi: 10.3389/fonc.2022.1024956 (PMC9684726; doi:10.3389/fonc.2022.1024956)
Supplement: Supplementary file 1 [file DataSheet_1.docx]

Supplementary Material

## Supplementary Figures

**
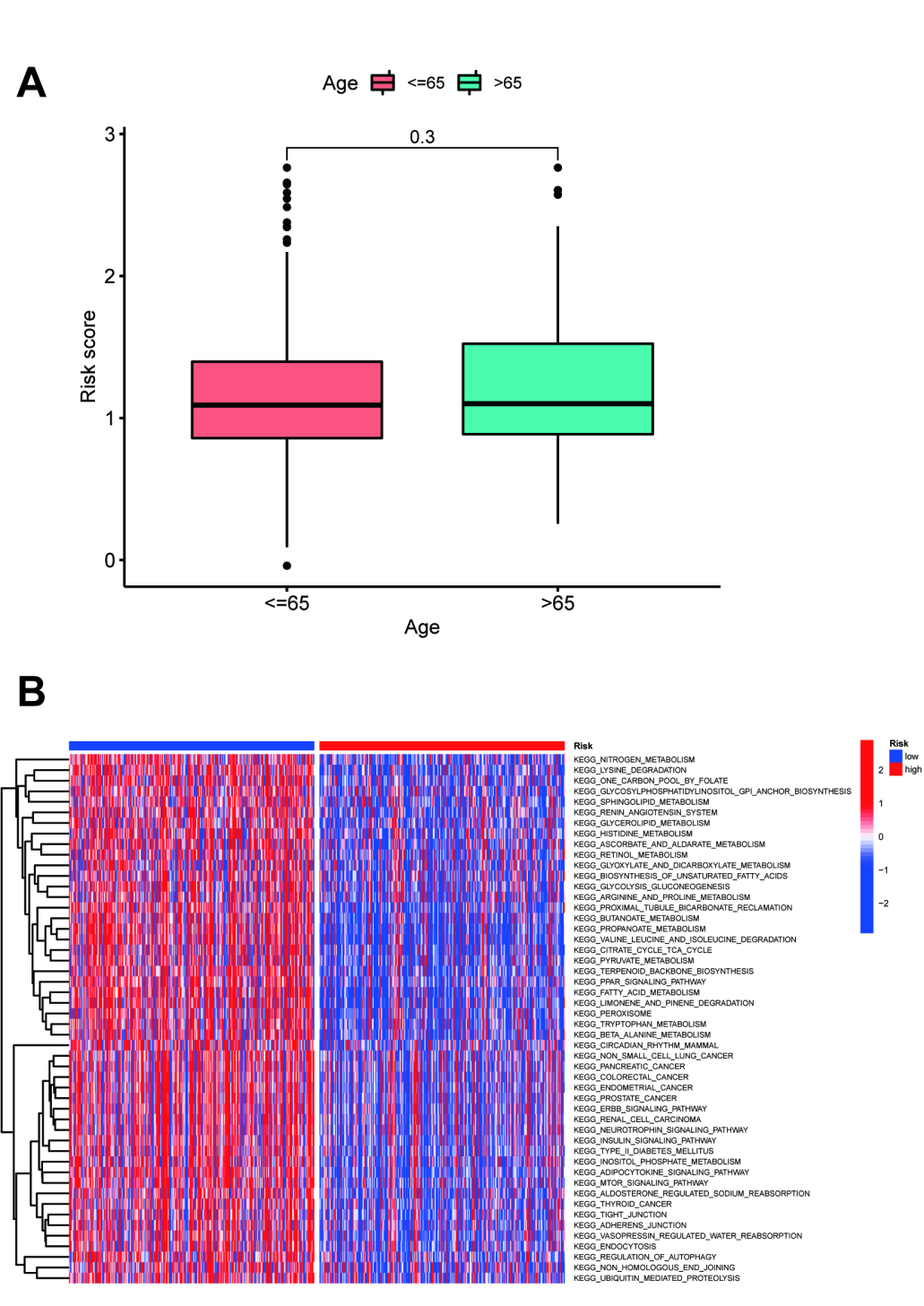
**

**Supplementary Figure 1. Clinical characteristics and GSVA enrichment analysis in high and low risk groups. A.** The heatmap of GSVA enrichment between low- and high-risk score groups. **B.** The relationship of clinicopathological features that’s Age with risk scores.

**
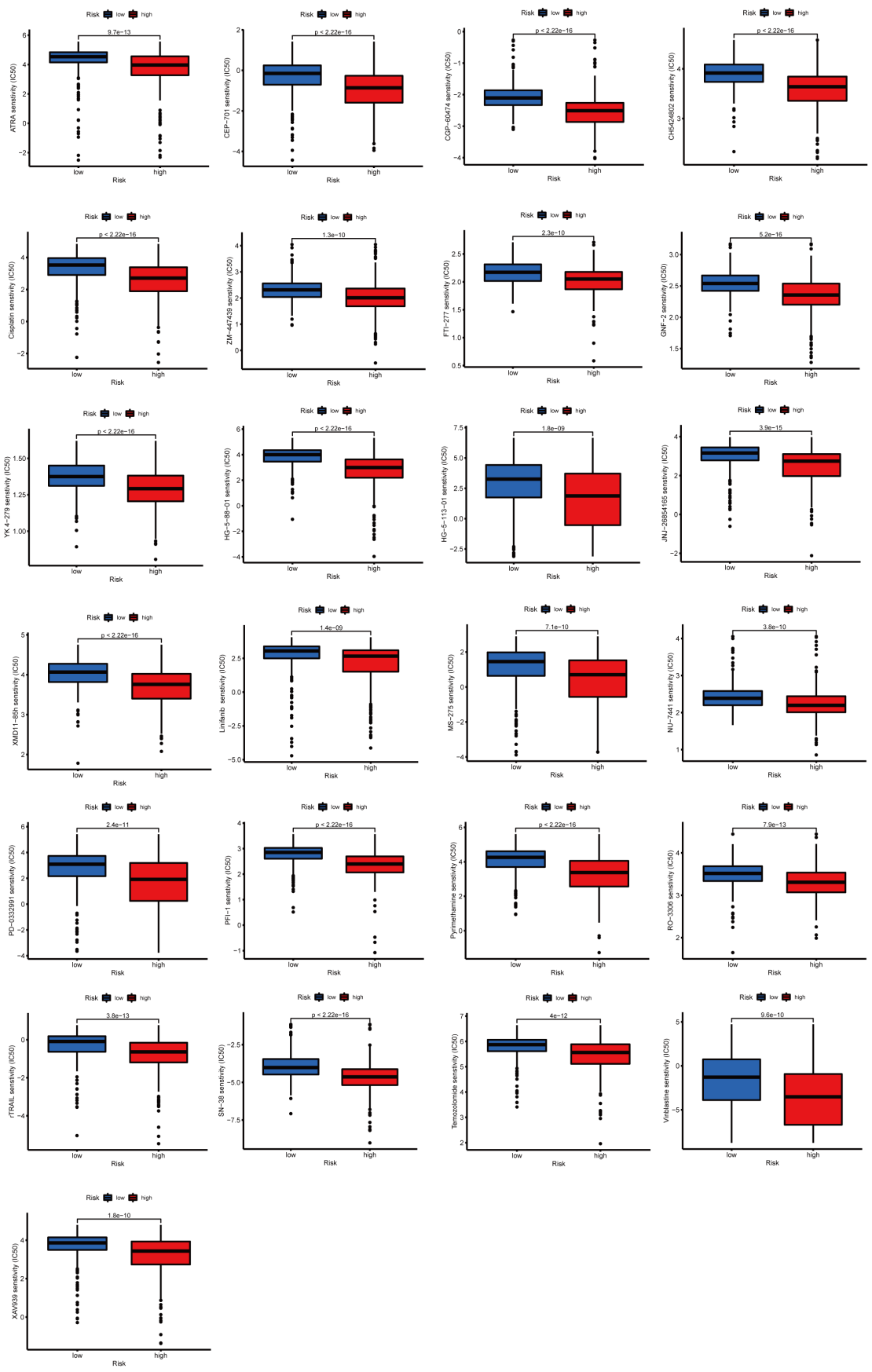
**

**Supplementary Figure 2.** The IC50 values of chemotherapy drugs were lower in the high-risk score group.


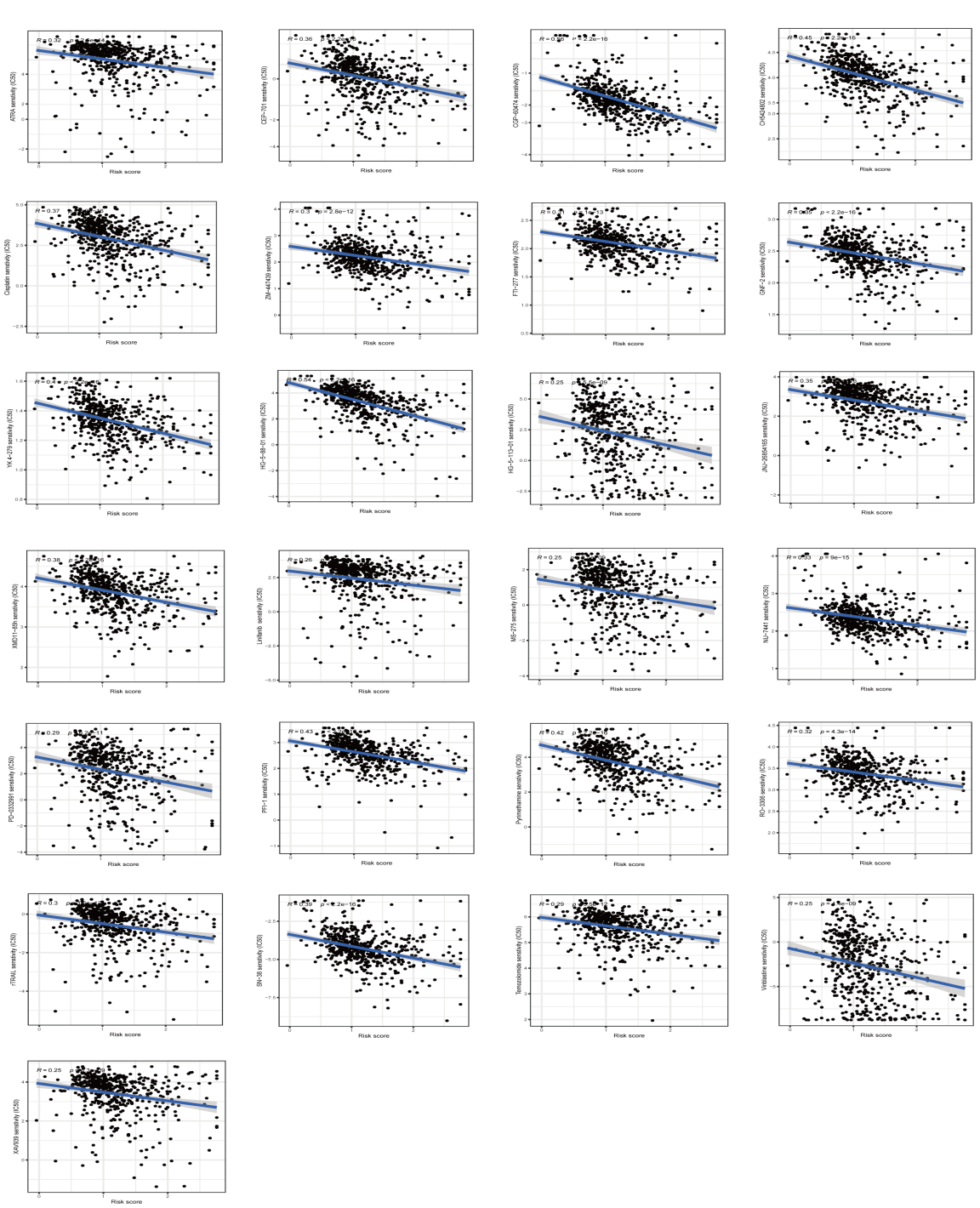


**Supplementary Figure 3.** Patients' risk scores were negatively correlated with chemotherapy resistance.


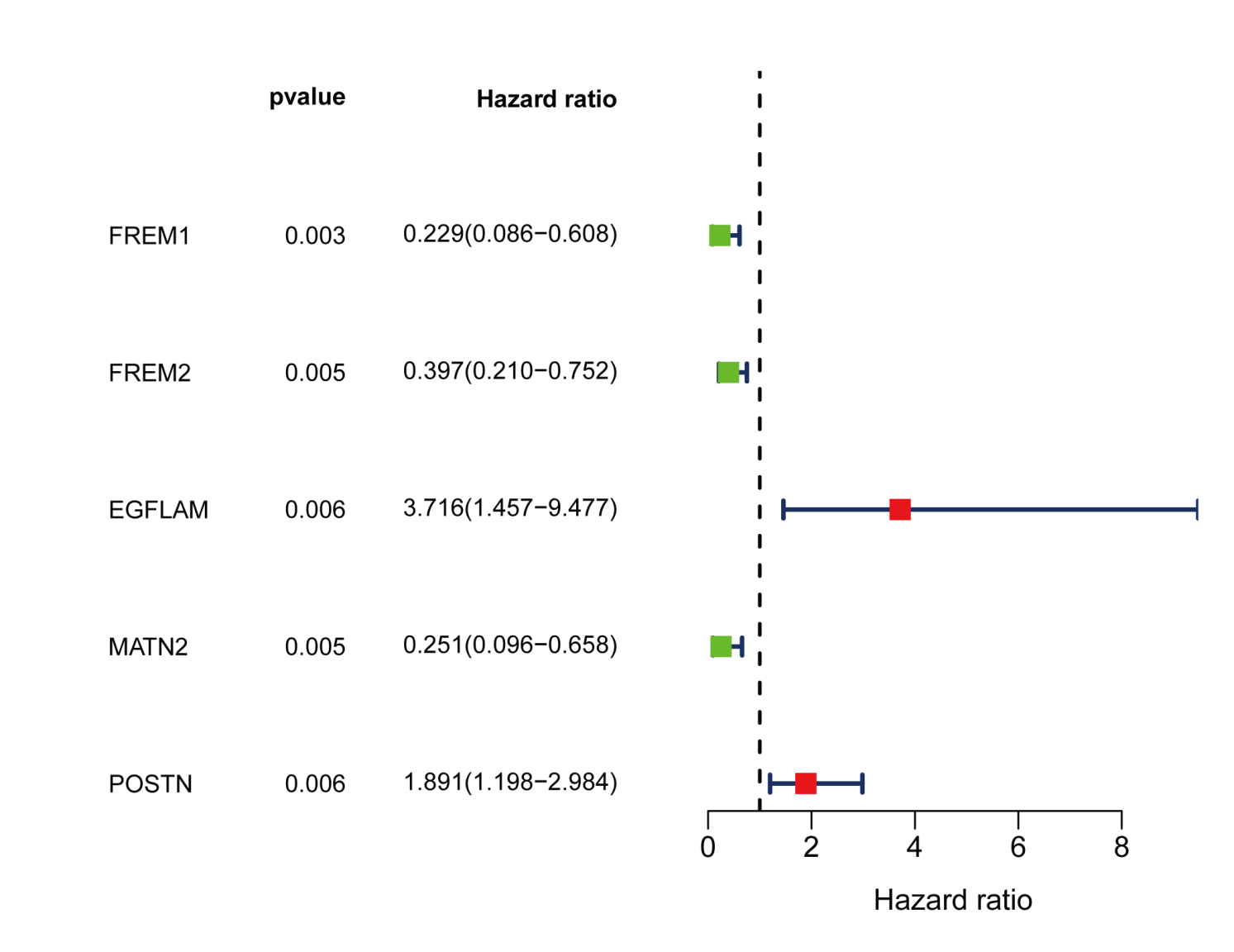


**Supplementary Figure 4.** The forest plot illustrated uni-Cox-reg analysis of the association of 5 BMGs with prognosis.
