# Supplementary material for: Characterization of the basement membrane in kidney renal clear cell carcinoma to guide clinical therapy
Source: Front Oncol. 2022 Nov 10;12:1024956. doi: 10.3389/fonc.2022.1024956 (PMC9684726; doi:10.3389/fonc.2022.1024956)
Supplement: Supplementary file 2 [file DataSheet_2.docx]

**Supplementary Table1: Basement membrane differentially expressed genes.**

| gene | conMean | treatMean | logFC | pValue | fdr |
| --- | --- | --- | --- | --- | --- |
| ACAN | 0.759415278 | 4.676292976 | 2.622404391 | 1.83E-24 | 6.30E-24 |
| ADAMTS10 | 0.757093056 | 3.537613678 | 2.224233968 | 2.05E-27 | 9.06E-27 |
| ADAMTS18 | 0.136593056 | 0.383836044 | 1.490606057 | 9.75E-13 | 1.86E-12 |
| ADAMTS2 | 1.060965278 | 3.987259335 | 1.910020001 | 2.25E-25 | 8.43E-25 |
| ANG | 2.727515278 | 8.353259335 | 1.614751951 | 3.53E-24 | 1.15E-23 |
| BGN | 219.5006444 | 461.9756113 | 1.073591515 | 1.03E-11 | 1.87E-11 |
| COL4A1 | 28.18283194 | 103.7135662 | 1.879716124 | 7.85E-29 | 4.22E-28 |
| COL4A2 | 22.59543333 | 65.29636081 | 1.530971363 | 3.39E-26 | 1.38E-25 |
| COL4A3 | 7.488261111 | 2.189678373 | -1.773911764 | 1.68E-32 | 1.39E-31 |
| COL4A4 | 11.72779583 | 2.707054529 | -2.11513604 | 1.64E-39 | 6.53E-38 |
| COL4A5 | 4.858676389 | 0.997842884 | -2.283678768 | 4.54E-39 | 1.51E-37 |
| COL4A6 | 2.693733333 | 0.134360444 | -4.325426669 | 1.09E-40 | 7.20E-39 |
| COL5A1 | 3.138726389 | 12.72181442 | 2.01905327 | 1.30E-18 | 3.12E-18 |
| COL6A1 | 29.10809167 | 63.10330702 | 1.116295355 | 3.11E-17 | 6.95E-17 |
| COL6A2 | 24.88211528 | 97.38614991 | 1.968607472 | 5.02E-28 | 2.32E-27 |
| COL6A3 | 4.956488889 | 12.8725353 | 1.376905825 | 4.63E-11 | 8.31E-11 |
| COL9A2 | 7.140754167 | 1.594613863 | -2.162869335 | 1.28E-33 | 1.34E-32 |
| COL9A3 | 0.507963889 | 0.205451571 | -1.305927576 | 3.42E-20 | 8.51E-20 |
| DAG1 | 35.09466389 | 15.83747542 | -1.147909307 | 1.26E-37 | 2.78E-36 |
| DCN | 39.60287083 | 6.786808318 | -2.544799844 | 3.57E-34 | 4.44E-33 |
| FBLN1 | 17.0853125 | 5.649411091 | -1.596584246 | 3.33E-25 | 1.20E-24 |
| FBLN5 | 43.24305139 | 13.2464597 | -1.706861497 | 8.53E-32 | 6.06E-31 |
| FGF9 | 9.110559722 | 1.063674492 | -3.09848297 | 1.39E-36 | 2.31E-35 |
| FN1 | 34.81148056 | 120.5603414 | 1.792120328 | 4.46E-22 | 1.29E-21 |
| FRAS1 | 6.685394444 | 2.866596118 | -1.221674029 | 1.19E-27 | 5.40E-27 |
| FREM1 | 6.329411111 | 0.607030314 | -3.382230808 | 1.19E-38 | 3.39E-37 |
| FREM2 | 5.280231944 | 1.721612939 | -1.616840478 | 4.32E-34 | 5.06E-33 |
| GPC3 | 48.64005417 | 3.513814048 | -3.791035085 | 1.84E-40 | 9.16E-39 |
| HMCN1 | 0.889429167 | 2.152858226 | 1.275301695 | 9.74E-16 | 2.02E-15 |
| ITGA2B | 0.170363889 | 0.434666728 | 1.351290101 | 0.032220318 | 0.036225103 |
| ITGB2 | 4.726654167 | 25.2493573 | 2.417355449 | 2.50E-34 | 3.31E-33 |
| ITGB6 | 20.61869028 | 3.360841959 | -2.617058086 | 5.90E-32 | 4.35E-31 |
| LAMA2 | 2.430968056 | 0.853311275 | -1.510386919 | 1.02E-21 | 2.83E-21 |
| LAMA4 | 2.529105556 | 16.1623403 | 2.675936958 | 9.02E-37 | 1.63E-35 |
| LAMC2 | 9.623940278 | 3.196492791 | -1.59013785 | 2.76E-22 | 8.09E-22 |
| MMP1 | 2.636790278 | 5.344215712 | 1.019195413 | 0.019083433 | 0.02170059 |
| MMP14 | 24.25034444 | 66.39933031 | 1.453163452 | 8.57E-29 | 4.49E-28 |
| MMP21 | 0.063583333 | 0.155843993 | 1.293381987 | 5.23E-09 | 8.54E-09 |
| P3H1 | 2.736523611 | 6.816068392 | 1.316595507 | 9.58E-34 | 1.06E-32 |
| PXDN | 4.330890278 | 13.8830268 | 1.680586614 | 4.89E-25 | 1.74E-24 |
| SERPINF1 | 10.179475 | 20.8424244 | 1.033859945 | 6.26E-06 | 8.59E-06 |
| SPARC | 166.0675208 | 602.4255131 | 1.859012929 | 1.14E-32 | 9.84E-32 |
| TGFBI | 33.31329861 | 235.5771791 | 2.82202967 | 3.62E-24 | 1.16E-23 |
| TLL1 | 0.8235875 | 2.504066543 | 1.604279062 | 8.97E-15 | 1.78E-14 |
| TNC | 32.46362639 | 15.37210961 | -1.078508998 | 1.18E-06 | 1.70E-06 |
| VCAN | 6.651572222 | 23.76189963 | 1.836882882 | 1.53E-17 | 3.51E-17 |
| ACHE | 0.604815278 | 1.509804067 | 1.319794849 | 0.002536573 | 0.003004632 |
| ADAMTS14 | 0.066315278 | 0.684268946 | 3.367150293 | 1.62E-30 | 9.49E-30 |
| ADAMTS15 | 6.628204167 | 1.666808503 | -1.991529677 | 2.29E-24 | 7.73E-24 |
| ADAMTS16 | 3.519559722 | 0.574472274 | -2.615085798 | 9.79E-31 | 6.44E-30 |
| ADAMTS19 | 0.257109722 | 0.013988355 | -4.200085955 | 5.01E-26 | 1.96E-25 |
| ADAMTS20 | 0.001072222 | 0.045936784 | 5.420974004 | 1.56E-26 | 6.48E-26 |
| ADAMTS4 | 2.366366667 | 9.479901294 | 2.002198402 | 5.00E-23 | 1.53E-22 |
| ADAMTS7 | 0.356377778 | 1.997913863 | 2.487015101 | 2.34E-36 | 3.59E-35 |
| ADAMTS8 | 0.877979167 | 0.375860628 | -1.223988906 | 1.25E-31 | 8.60E-31 |
| BCAN | 0.162544444 | 0.657933272 | 2.017107024 | 1.30E-26 | 5.51E-26 |
| CD44 | 7.322744444 | 19.00162237 | 1.375666248 | 1.02E-18 | 2.47E-18 |
| COL14A1 | 15.40383889 | 6.52099353 | -1.240126245 | 3.02E-14 | 5.96E-14 |
| COL15A1 | 8.751145833 | 23.8698647 | 1.447646555 | 1.03E-17 | 2.38E-17 |
| COL8A1 | 3.310843056 | 13.17587283 | 1.992628006 | 2.55E-24 | 8.45E-24 |
| CSPG4 | 2.842113889 | 19.29812144 | 2.763424144 | 1.77E-33 | 1.68E-32 |
| EGFLAM | 0.891381944 | 2.468175416 | 1.469329288 | 1.95E-15 | 3.99E-15 |
| EVA1C | 2.6321625 | 5.810718115 | 1.142467911 | 9.56E-23 | 2.88E-22 |
| FREM3 | 0.067031944 | 0.025156007 | -1.413945819 | 3.07E-21 | 8.26E-21 |
| GPC2 | 0.043031944 | 0.175848799 | 2.030855541 | 1.30E-24 | 4.54E-24 |
| GPC5 | 4.418809722 | 0.07759353 | -5.831577628 | 5.09E-43 | 1.01E-40 |
| HAPLN1 | 0.288006944 | 3.27683549 | 3.508127742 | 1.38E-30 | 8.30E-30 |
| HAPLN2 | 0.142375 | 0.324129575 | 1.186874822 | 3.57E-08 | 5.51E-08 |
| ITGA10 | 0.506975 | 1.156095564 | 1.189274145 | 1.04E-08 | 1.64E-08 |
| ITGA4 | 1.182563889 | 4.253771534 | 1.846824421 | 1.58E-28 | 8.05E-28 |
| ITGA5 | 7.957568056 | 31.08225009 | 1.965691451 | 5.25E-32 | 4.02E-31 |
| ITGAM | 1.252919444 | 5.141182625 | 2.0368066 | 1.00E-30 | 6.44E-30 |
| ITGAX | 0.567368056 | 5.573861368 | 3.29632029 | 2.55E-38 | 6.33E-37 |
| LAD1 | 16.44293056 | 2.654174677 | -2.631132222 | 1.97E-37 | 3.91E-36 |
| LAMB4 | 0.616293056 | 0.230308133 | -1.420051176 | 1.53E-33 | 1.52E-32 |
| LOXL2 | 3.307356944 | 20.96690074 | 2.664362962 | 3.92E-33 | 3.55E-32 |
| LOXL4 | 8.260886111 | 3.675176155 | -1.16848314 | 7.62E-11 | 1.32E-10 |
| LUM | 84.59650833 | 34.38096728 | -1.298987984 | 6.85E-18 | 1.60E-17 |
| MATN1 | 0.022598611 | 0.074705545 | 1.724981227 | 3.37E-23 | 1.07E-22 |
| MATN2 | 15.40010972 | 7.11484732 | -1.114035927 | 1.30E-25 | 4.97E-25 |
| MEGF6 | 1.422291667 | 3.687867283 | 1.374569392 | 6.00E-16 | 1.28E-15 |
| MEP1A | 0.007488889 | 0.055385213 | 2.886677249 | 2.58E-25 | 9.51E-25 |
| MEP1B | 0.014280556 | 0.049963586 | 1.806824919 | 2.37E-08 | 3.69E-08 |
| MMP17 | 0.107366667 | 0.442555823 | 2.043313284 | 8.08E-08 | 1.24E-07 |
| MMP7 | 116.8238292 | 57.91901664 | -1.012225565 | 7.37E-11 | 1.29E-10 |
| NELL1 | 9.371048611 | 0.162809612 | -5.846952713 | 4.62E-42 | 4.60E-40 |
| NELL2 | 0.273566667 | 0.73722403 | 1.430210643 | 1.11E-09 | 1.87E-09 |
| NID1 | 17.65227222 | 46.37133235 | 1.393379278 | 3.79E-23 | 1.18E-22 |
| NID2 | 3.450475 | 6.930003512 | 1.006061103 | 9.10E-09 | 1.46E-08 |
| NPNT | 36.93389861 | 9.452233272 | -1.966218417 | 1.07E-30 | 6.66E-30 |
| NTN4 | 91.0013625 | 28.94002348 | -1.652822054 | 2.56E-32 | 2.04E-31 |
| OGN | 3.178719444 | 1.442124399 | -1.140250071 | 6.65E-16 | 1.39E-15 |
| POSTN | 8.3096625 | 25.19117449 | 1.600056599 | 9.30E-13 | 1.80E-12 |
| SEMA3B | 12.35799861 | 4.859033457 | -1.346703845 | 2.22E-28 | 1.10E-27 |
| SLIT1 | 0.035436111 | 0.077607763 | 1.130980692 | 5.82E-15 | 1.17E-14 |
| SLIT2 | 4.204031944 | 1.80920573 | -1.216417161 | 7.37E-14 | 1.44E-13 |
| SPARCL1 | 57.94417361 | 178.0537113 | 1.619576999 | 4.03E-21 | 1.07E-20 |
| SPOCK2 | 43.86384167 | 21.72941719 | -1.013382691 | 5.45E-17 | 1.19E-16 |
| SPON2 | 4.778336111 | 15.75248595 | 1.720999281 | 3.89E-17 | 8.60E-17 |
| TENM1 | 0.370611111 | 2.178007763 | 2.555031057 | 6.93E-27 | 3.00E-26 |
| THBS2 | 3.866925 | 9.496812015 | 1.296256513 | 4.24E-05 | 5.63E-05 |
| THBS4 | 0.375773611 | 1.626692052 | 2.1140055 | 0.000867651 | 0.001065818 |
| TIMP1 | 82.12318472 | 329.5530963 | 2.004649444 | 4.10E-26 | 1.63E-25 |
| UNC5A | 0.059651389 | 0.627016821 | 3.393876512 | 4.77E-28 | 2.26E-27 |
| UNC5B | 5.385597222 | 18.84313512 | 1.806860778 | 2.78E-28 | 1.35E-27 |
| VWA1 | 14.58835417 | 36.33150388 | 1.316403955 | 6.47E-22 | 1.81E-21 |

**Supplementary Table 2: Following formula to calculate the risk score.**

| Gene | Coef |
| --- | --- |
| COL4A4 | -0.000332379 |
| FREM2 | -0.045328401 |
| P3H1 | 0.286241547 |
| SERPINF1 | 0.009254363 |
| TLL1 | -0.003926575 |
| ACHE | 0.10443199 |
| ADAMTS14 | 0.192192784 |
| CD44 | 0.048163835 |
| MMP17 | 0.088010757 |
| NPNT | -0.121725612 |

**Supplementary Table 3: The correlation of immune cells calculated on different platforms.**

| Immune | cor | pvalue |
| --- | --- | --- |
| T cell CD4+_TIMER | 0.188607128 | 1.19E-05 |
| B cell naive_CIBERSORT | -0.18857645 | 1.19E-05 |
| B cell memory_CIBERSORT | 0.192632044 | 7.66E-06 |
| T cell CD4+ memory activated_CIBERSORT | 0.188679521 | 1.18E-05 |
| T cell follicular helper_CIBERSORT | 0.263560195 | 6.65E-10 |
| T cell regulatory (Tregs)_CIBERSORT | 0.390622977 | 7.74E-21 |
| Monocyte_CIBERSORT | -0.211259492 | 8.79E-07 |
| Macrophage M0_CIBERSORT | 0.327105063 | 9.87E-15 |
| Mast cell activated_CIBERSORT | -0.175745618 | 4.59E-05 |
| B cell memory_CIBERSORT-ABS | 0.194712146 | 6.07E-06 |
| T cell CD8+_CIBERSORT-ABS | 0.179664103 | 3.07E-05 |
| T cell CD4+ memory activated_CIBERSORT-ABS | 0.188872988 | 1.16E-05 |
| T cell follicular helper_CIBERSORT-ABS | 0.289959419 | 9.15E-12 |
| T cell regulatory (Tregs)_CIBERSORT-ABS | 0.42119149 | 2.73E-24 |
| Macrophage M0_CIBERSORT-ABS | 0.333652711 | 2.67E-15 |
| Macrophage M1_QUANTISEQ | 0.406378027 | 1.43E-22 |
| Macrophage M2_QUANTISEQ | 0.209438727 | 1.10E-06 |
| Neutrophil_QUANTISEQ | -0.402941073 | 3.47E-22 |
| T cell CD4+ (non-regulatory)_QUANTISEQ | -0.24711253 | 7.65E-09 |
| T cell CD8+_QUANTISEQ | 0.263135161 | 7.10E-10 |
| T cell regulatory (Tregs)_QUANTISEQ | 0.288464101 | 1.18E-11 |
| T cell CD8+_MCPCOUNTER | 0.172606167 | 6.28E-05 |
| Neutrophil_MCPCOUNTER | -0.351877052 | 5.96E-17 |
| Endothelial cell_MCPCOUNTER | -0.221657663 | 2.41E-07 |
| Cancer associated fibroblast_MCPCOUNTER | 0.396282345 | 1.89E-21 |
| Myeloid dendritic cell activated_XCELL | 0.239987124 | 2.09E-08 |
| B cell_XCELL | 0.268605068 | 3.04E-10 |
| T cell CD4+ naive_XCELL | 0.245543984 | 9.57E-09 |
| T cell CD4+ effector memory_XCELL | 0.297537141 | 2.45E-12 |
| T cell CD8+_XCELL | 0.19952439 | 3.52E-06 |
| T cell CD8+ effector memory_XCELL | 0.244173811 | 1.16E-08 |
| Class-switched memory B cell_XCELL | 0.209268735 | 1.12E-06 |
| Common lymphoid progenitor_XCELL | -0.238516972 | 2.56E-08 |
| Myeloid dendritic cell_XCELL | 0.247189657 | 7.56E-09 |
| Hematopoietic stem cell_XCELL | -0.305958911 | 5.43E-13 |
| Macrophage_XCELL | 0.236078717 | 3.58E-08 |
| Macrophage M1_XCELL | 0.348210986 | 1.31E-16 |
| Monocyte_XCELL | 0.289508127 | 9.88E-12 |
| B cell naive_XCELL | 0.220902834 | 2.65E-07 |
| T cell NK_XCELL | 0.560279997 | 2.69E-45 |
| Plasmacytoid dendritic cell_XCELL | 0.231891802 | 6.30E-08 |
| T cell CD4+ Th1_XCELL | 0.332986719 | 3.06E-15 |
| T cell CD4+ Th2_XCELL | 0.199384107 | 3.58E-06 |
| immune score_XCELL | 0.293875893 | 4.66E-12 |
| microenvironment score_XCELL | 0.260142062 | 1.12E-09 |
| Cancer associated fibroblast_EPIC | 0.439405932 | 1.60E-26 |
| T cell CD4+_EPIC | -0.341744031 | 5.09E-16 |
| T cell CD8+_EPIC | -0.260486397 | 1.06E-09 |
| Endothelial cell_EPIC | -0.245423688 | 9.73E-09 |
| Macrophage_EPIC | 0.377489364 | 1.84E-19 |
| NK cell_EPIC | 0.347042921 | 1.67E-16 |
